# AI and ML-based risk assessment of chemicals: predicting carcinogenic risk from chemical-induced genomic instability

**DOI:** 10.3389/ftox.2024.1461587

**Published:** 2024-11-26

**Authors:** Ajay Vikram Singh, Preeti Bhardwaj, Peter Laux, Prachi Pradeep, Madleen Busse, Andreas Luch, Akihiko Hirose, Christopher J. Osgood, Michael W. Stacey

**Affiliations:** ^1^ Department of Chemical and Product Safety, German Federal Institute for Risk Assessment (BfR), Berlin, Germany; ^2^ Department of Biological Safety, German Federal Institute for Risk Assessment (BfR), Berlin, Germany; ^3^ Chemicals Evaluation and Research Institute, Tokyo, Japan; ^4^ Department of Biological Sciences, Old Dominion University, Norfolk, VA, United States; ^5^ Frank Reidy Research Center for Bioelectrics, Old Dominion University, Norfolk, VA, United States

**Keywords:** artificial intelligence (AI), machine learning (ML), genomic instability, chemical risk assessment, clastogen-induced carcinogenesis

## Abstract

Chemical risk assessment plays a pivotal role in safeguarding public health and environmental safety by evaluating the potential hazards and risks associated with chemical exposures. In recent years, the convergence of artificial intelligence (AI), machine learning (ML), and omics technologies has revolutionized the field of chemical risk assessment, offering new insights into toxicity mechanisms, predictive modeling, and risk management strategies. This perspective review explores the synergistic potential of AI/ML and omics in deciphering clastogen-induced genomic instability for carcinogenic risk prediction. We provide an overview of key findings, challenges, and opportunities in integrating AI/ML and omics technologies for chemical risk assessment, highlighting successful applications and case studies across diverse sectors. From predicting genotoxicity and mutagenicity to elucidating molecular pathways underlying carcinogenesis, integrative approaches offer a comprehensive framework for understanding chemical exposures and mitigating associated health risks. Future perspectives for advancing chemical risk assessment and cancer prevention through data integration, advanced machine learning techniques, translational research, and policy implementation are discussed. By implementing the predictive capabilities of AI/ML and omics technologies, researchers and policymakers can enhance public health protection, inform regulatory decisions, and promote sustainable development for a healthier future.

## 1 Introduction

Chemical exposure is ubiquitous in modern society, arising from various sources such as industrial processes, consumer products, agriculture, and environmental pollutant (Rai et al., 2023b). While many chemicals serve essential purposes in daily life, some possess inherent toxic properties that can pose significant risks to human health. Among these are chemical clastogens, substances capable of inducing DNA damage and genomic instability (Goel et al., 2023). Chemical clastogens exert their deleterious effects by disrupting the integrity of the genome, the complete set of an organism’s genetic material encoded in DNA. These substances can induce a wide range of DNA lesions, including single and double-strand breaks, DNA adducts, chromosomal rearrangements, and point mutations (Bignold, 2009). Genomic instability, characterized by an increased frequency of such genetic aberrations, is a hallmark feature of cancer and other genetic diseases^4^. Understanding the mechanisms by which chemical clastogens cause genomic instability is crucial for elucidating their carcinogenic potential and devising effective strategies for risk assessment and mitigation (Singh et al., 2020a). By unraveling the complex interplay between chemical exposures and DNA damage response pathways, researchers can identify biomarkers of exposure, predict carcinogenic outcomes, and develop targeted interventions to reduce cancer risk (Bolzán, 2020).

### 1.1 Importance of chemical risk assessment for potential carcinogen classification

Chemical risk assessment plays a pivotal role in cancer prevention efforts by identifying and characterizing the hazards associated with exposure to potentially carcinogenic substances. Regulatory agencies worldwide rely on risk assessment frameworks to evaluate the safety of chemicals used in consumer products, food additives, industrial processes, and environmental contaminants (Jovanović et al., 2023). Through rigorous toxicological testing and epidemiological studies, risk assessors determine the likelihood and severity of adverse health effects posed by specific chemicals and establish exposure limits and regulatory standards to protect public health (Goodman et al., 2020).

Effective chemical risk assessment requires a multidisciplinary approach that integrates data from diverse scientific disciplines, including toxicology, epidemiology, molecular biology, and computational modeling (Nayar). By systematically evaluating the hazards, exposures, and risks associated with chemical substances, risk assessors can inform regulatory decisions, guide risk management strategies, and prioritize resources for further research and monitoring (Butnariu and Bonciu, 2022).

### 1.2 Role of AI/ML and omics technologies in enhancing risk assessment

Advances in artificial intelligence (AI), machine learning (ML), and omics technologies are revolutionizing the field of chemical risk assessment by providing powerful tools for data analysis, predictive modeling, and biomarker discovery (Ojji, 2024). AI/ML algorithms can analyze vast datasets comprising chemical structures, toxicity profiles, and biological responses to identify patterns, correlations, and predictive relationships that may not be apparent through traditional statistical methods (Khadela et al., 2023).

Omics technologies, including genomics, transcriptomics, proteomics, metabolomics and cytomics, enable comprehensive molecular profiling of biological systems in response to chemical exposures (Del Giudice et al., 2023). By capturing global changes in gene expression, protein abundance, and metabolite levels, omics approaches provide insights into the molecular mechanisms underlying chemical toxicity and facilitate the identification of biomarkers indicative of adverse health effects (Singh et al., 2021a). The integration of AI/ML and omics technologies holds immense promise for enhancing the predictive accuracy, efficiency, and scalability of chemical risk assessment. By leveraging computational models trained on large-scale omics datasets, researchers can predict the toxicological properties of chemical compounds, prioritize compounds for further testing, and extrapolate toxicity data across chemical classes and species (Yang et al., 2018). Additionally, AI-driven approaches can facilitate the identification of novel biomarkers and molecular signatures of chemical exposure that may serve as early indicators of adverse health outcomes (Scott et al., 2023).

The convergence of AI/ML and omics technologies represents a paradigm shift in chemical risk assessment, enabling a more holistic and data-driven approach to understanding chemical toxicity, identifying hazards, and safeguarding public health. By harnessing the power of computational modeling, high-throughput screening, and molecular profiling, researchers can accelerate the pace of discovery, improve risk prediction accuracy, and ultimately reduce the burden of cancer and other diseases associated with chemical exposures (Vindman et al., 2024). This perspective review provides a detailed overview on chemical clastogens and their role in genomic instability, the importance of chemical risk assessment in cancer prevention, and the potential of AI/ML and omics technologies in enhancing risk assessment.

### 1.3 AI applications in cancer research: Insights from TCGA and EPA’s OncoLogic AI tool

The application of artificial intelligence (AI) in cancer research has revolutionized the way we interpret large-scale genomic data, with The Cancer Genome Atlas (TCGA) serving as a cornerstone for such efforts. AI algorithms, including machine learning (ML) and deep learning models, have been successfully applied to TCGA datasets, leading to significant advancements in cancer subtype classification, biomarker discovery, and the understanding of cancer progression (Deepa et al., 2023; Deepa et al., 2023).

For example, AI-based approaches have been instrumental in integrating multi-omics data from TCGA to identify novel cancer subtypes, such as in glioblastoma and breast cancer, where molecular subtyping has led to more personalized therapeutic strategies (He et al., 2023). Additionally, AI has enhanced the prediction of patient outcomes by identifying prognostic biomarkers that are often missed by traditional statistical methods (Huang et al., 2020). While the application of AI to TCGA has yielded many successes, it is not without limitations. One key challenge is the heterogeneity of cancer, both within a tumor and across patients. AI models trained on TCGA data sometimes struggle with this variability, which can affect the generalizability of findings. Moreover, while AI excels at identifying correlations and patterns in large datasets, establishing causal relationships between molecular alterations and clinical outcomes remains challenging. Nonetheless, continued advancements in AI and the integration of more comprehensive datasets will further enhance our ability to uncover actionable insights from TCGA (Davis et al., 2018).

The EPA’s OncoLogic^®^ is one of the most established predictive tools for regulatory applications in carcinogenic risk assessment. Developed to estimate the carcinogenic potential of chemical compounds, OncoLogic^®^ employs a structured approach that integrates expert judgment with encoded empirical data. According to Benigni et al. (2012), the system assesses multiple factors, primarily focusing on chemical structure and known biological activity, and uses structural alerts based on extensive toxicological knowledge (Benigni et al., 2012). However, it does not inherently incorporate epidemiological evidence unless this has been specifically encoded within its rules. OncoLogic^®^ functions primarily as a class-based system, and while it excels in pre-manufacture notifications (PMN) for novel chemical substances, it is not designed for pesticide registrations, which require empirical testing. The system has been validated for its predictive accuracy in various chemical classes, highlighting its utility in evaluating industrial chemicals and its evolving role in regulatory risk assessments (Benigni et al., 2012). This highlights OncoLogic^®^ as a key tool in informing regulatory decisions, with continued advancements enhancing its effectiveness and transparency in carcinogenicity prediction.

### 1.4 Chemical clastogens and their mechanisms of carcinogenesis

**FIGURE 1:**
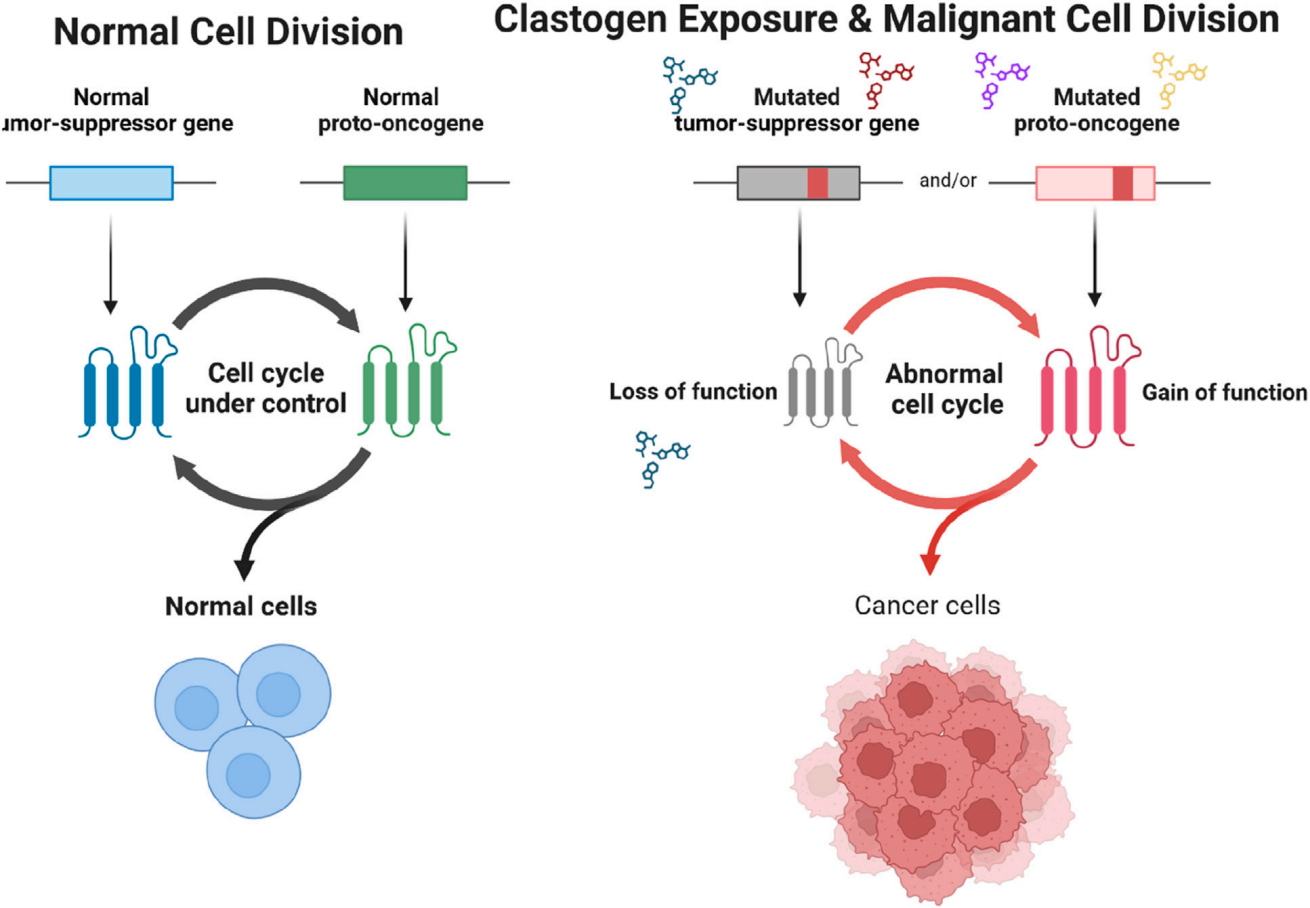
Comparison of Normal Cell Division and Cancerous Cell Division Following Clastogen Exposure. The diagram contrasts normal cell division with malignant cell division triggered by clastogen exposure. In normal cells, the cell cycle is regulated by functioning tumor-suppressor genes and proto-oncogenes, maintaining controlled cell growth. Exposure to clastogens can lead to mutations in tumor-suppressor genes and proto-oncogenes. Mutated tumor-suppressor genes lose their function, while mutated proto-oncogenes gain oncogenic functions, resulting in an abnormal cell cycle. This dysregulation promotes the proliferation of cancer cells.

Chemical clastogens encompass a diverse array of substances that possess the ability to induce DNA damage and genomic instability, thereby increasing the risk of cancer and other genetic disorders changing fate of normal cell division to uncontrolled cancerous growth as shown below in Figure 1 (Singh A. K. et al., 2024). Understanding the various types of chemical clastogens, their mechanisms of action, and the specific mutations they induce is essential for elucidating their carcinogenic potential and devising effective strategies for risk assessment and mitigation (Bashetti et al., 2024). Chemical clastogens can be broadly classified into several categories based on their chemical structure, mode of action, and biological effects (Zeliger, 2022). Some of the most common types of chemical clastogens include:

1. Benzene Derivatives: Benzene and its derivatives, such as toluene and xylene, are organic solvents widely used in industrial processes, fuel production, and consumer products. These compounds can undergo metabolic activation to form reactive intermediates that covalently bind to DNA, leading to the formation of DNA adducts and chromosomal aberrations (Barton, 2023).
2. Polycyclic Aromatic Hydrocarbons (PAHs): PAHs are a group of environmental pollutants formed during incomplete combustion of organic materials, such as fossil fuels, tobacco smoke, and grilled meats. Benzo [a]pyrene, a prototypical PAH, can intercalate into DNA and undergo metabolic activation to form reactive metabolites that induce DNA adducts and cause chromosomal damage (Beland et al., 2001).
3. Formaldehyde: formaldehyde is a ubiquitous industrial chemical used in the production of resins, plastics, and disinfectants. It can react with DNA and proteins to form crosslinks, DNA-protein adducts, and DNA single-strand breaks, leading to chromosomal instability and genotoxicity (Singh et al., 2021b; Rai et al., 2023a).
4. Alkylating Agents: Alkylating agents, such as nitrogen mustards and ethyl methanesulfonate (EMS), are chemical compounds that alkylate DNA bases, leading to the formation of DNA adducts and DNA crosslinks. These compounds can induce a wide range of genetic mutations, including point mutations, deletions, and insertions (Kim et al., 2016).
5. Aflatoxins: Aflatoxins are fungal metabolites produced by Aspergillus species that contaminate food crops such as peanuts, corn, and grains. Aflatoxin B1 (AFB1), the most potent aflatoxin, can undergo metabolic activation to form DNA adducts that lead to the formation of G to T transversions and hepatocellular carcinoma (HCC) (Zahra et al., 2023).

### 1.5 Mechanisms of action leading to genomic instability

The mechanisms by which chemical clastogens induce genomic instability are diverse and complex, involving multiple pathways and molecular targets within the cell. Some of the key mechanisms of action leading to genomic instability include:

1. DNA Damage: Chemical clastogens can directly damage DNA molecules by inducing single and double-strand breaks, DNA adduct formation, crosslinking, and base modifications. These DNA lesions can interfere with DNA replication, transcription, and repair processes, leading to genomic instability and mutagenesis (Shukla et al., 2021).
2. Cellular Metabolism: Many chemical clastogens require metabolic activation by cellular enzymes to exert their genotoxic effects. Metabolic activation can lead to the formation of reactive intermediates that covalently bind to DNA, proteins, and other cellular macromolecules, disrupting their normal functions and inducing DNA damage (de Oliveira et al., 2021).
3. Oxidative Stress: Some chemical clastogens, such as ionizing radiation and certain chemicals, can generate reactive oxygen species (ROS) within the cell. ROS can oxidize DNA bases, induce DNA strand breaks, and alter cellular signaling pathways involved in DNA repair and apoptosis, contributing to genomic instability and carcinogenesis (Jomova et al., 2023).

### 1.6 Specific mutations and their consequences

The genotoxic effects of chemical clastogens can manifest as specific types of mutations in the genome, each with distinct consequences for cellular function and organismal health (Singh et al., 2020b). Some of the most common types of mutations induced by chemical clastogens include:

1. Point Mutations: Chemical clastogens can induce point mutations by altering the nucleotide sequence of DNA. This can result in the substitution of one nucleotide for another (e.g., G to T transversions), insertion or deletion of nucleotides (indels), or other base-pair substitutions, leading to changes in protein structure and function (Beal et al., 2020).
2. Chromosomal Aberrations: Chemical clastogens can cause structural changes in chromosomes, such as chromosomal translocations, inversions, deletions, and duplications. These chromosomal aberrations can disrupt normal gene expression patterns, alter cellular signaling pathways, and contribute to oncogenic transformation (Bignold, 2009; Mishima, 2017).
3. Aneuploidy: Aneuploidy, the gain or loss of whole chromosomes, can result from errors in chromosome segregation during cell division induced by chemical clastogens. Aneuploidy can disrupt cellular homeostasis, impair cell viability, and promote tumorigenesis by altering gene dosage and expression levels (Xu and Adler, 1990).
4. Activation of proto-oncogenes: Mutations in proto-oncogenes, such as ras, myc, and erbB2, can lead to their constitutive activation, converting them into oncogenes that promote uncontrolled cell proliferation and cancer (Botezatu et al., 2016).
5. Inactivation of tumor suppressor genes: Mutations in tumor suppressor genes, like p53, Rb, BRCA1/2, and PTEN, can result in the loss of their normal function, allowing cells with damaged DNA to continue dividing and evade apoptosis (Wang et al., 2019).
6. Genomic instability and chromosomal abnormalities: Chromosomal aberrations, such as gains, losses, and rearrangements, can lead to aneuploidy and further genomic instability, which are hallmarks of cancer development (Sansregret et al., 2018).

Understanding the specific mutations induced by chemical clastogens and their downstream consequences is essential for elucidating the mechanisms of carcinogenesis and designing effective strategies for cancer prevention and intervention (Singh et al., 2020b). The DNA damage and chromosomal aberrations induced by chemical clastogens can result in significant consequences for cellular function (Barnes et al., 2018).

### 1.7 Activation and deactivation of genes by clastogens: fate of proto-oncogenes and tumor suppressor genes

Chemical clastogens have the potential to modulate the expression and activity of genes involved in cell growth, differentiation, and survival, thereby influencing carcinogenesis and disease progression. Understanding how clastogens interact with proto-oncogenes and tumor suppressor genes, as well as the molecular mechanisms underlying gene regulation, is crucial for deciphering their oncogenic effects and developing targeted therapeutic strategies. Proto-oncogenes are a class of genes that regulate cell proliferation, survival, and differentiation under normal physiological conditions (Shortt and Johnstone, 2012). However, dysregulation or aberrant activation of proto-oncogenes can promote uncontrolled cell growth and contribute to tumorigenesis (Okabe and Kaneda, 2021). Chemical clastogens can activate proto-oncogenes through various mechanisms (Lo, 2002), including:

1. Chromosomal Translocations: Clastogens can induce chromosomal translocations that juxtapose proto-oncogenes with highly active regulatory elements, leading to their constitutive activation. For example, the t (8; 14) translocation involving the c-Myc proto-oncogene is commonly associated with lymphoid malignancies such as Burkitt lymphoma (O’Connor, 2008).
2. Point Mutations: Chemical clastogens can introduce specific point mutations in proto-oncogenes that enhance their oncogenic activity. For instance, mutations in the RAS family of proto-oncogenes can lead to constitutive activation of RAS signaling pathways, promoting cell proliferation and survival (Prior et al., 2012).

Tumor suppressor genes, on the other hand, encode proteins that inhibit cell growth and proliferation or promote apoptosis in response to cellular stress or DNA damage. Loss or inactivation of tumor suppressor genes can retract these growth inhibitory signals and predispose cells to malignant transformation (Chow, 2010). Chemical clastogens can deactivate tumor suppressor genes through mechanisms such as point mutations and seletions (Langie et al., 2015). Clastogens can introduce mutations or deletions in tumor suppressor genes, compromising their function and abrogating their tumor suppressive activities. For example, inactivating mutations in the TP53 tumor suppressor gene are commonly observed in various human cancers, allowing cells to evade apoptosis and proliferate uncontrollably (Rivlin et al., 2011). Epigenetic silencing is further linked with ON/OFF mechanism induced by clastogens. Chemical clastogens can induce epigenetic modifications, such as DNA methylation and histone modifications, that silence the expression of tumor suppressor genes (Sabarwal et al., 2018). This epigenetic silencing can occur through aberrant recruitment of chromatin-modifying enzymes or alterations in DNA methylation patterns, leading to transcriptional repression of tumor suppressor genes (Spatari et al., 2021). Understanding the interplay between chemical clastogens and proto-oncogenes/tumor suppressor genes is essential for elucidating their role in carcinogenesis and identifying potential targets for therapeutic intervention.

### 1.8 Epigenetic hallmarks of cancer

Recent research has highlighted the critical role of epigenetic mechanisms in cancer development, with growing evidence suggesting that alterations in the epigenome can drive cancer independently of genetic mutations. The updated hallmarks of cancer, as outlined by the AACR, emphasize how disruptions in normal epigenetic regulation contribute to carcinogenesis. These include DNA methylation, histone modification, and chromatin remodeling, which collectively alter gene expression patterns without changing the DNA sequence itself (Gibney and Nolan, 2010). Epigenetic modifications can lead to the silencing of tumor suppressor genes or the activation of oncogenes, thus promoting tumor growth and metastasis. For instance, hypermethylation of promoter regions in key tumor suppressor genes such as p16INK4a and BRCA1 has been observed in various cancers, leading to their functional inactivation. Furthermore, global hypomethylation can result in chromosomal instability, another hallmark of cancer (Kawano et al., 2014).

Importantly, epigenetic mechanisms alone have been shown to induce cancer. Several studies, including recent work on hematological malignancies, have demonstrated that aberrant epigenetic regulation can be sufficient to initiate and drive tumorigenesis (Sharma et al., 2010). Understanding these epigenetic hallmarks offers new avenues for cancer prevention and treatment, as epigenetic alterations are reversible, making them attractive targets for therapeutic intervention using agents such as DNA methyltransferase inhibitors (e.g., decitabine) and histone deacetylase inhibitors (e.g., vorinostat) (Cheng et al., 2019).

### 1.9 Molecular mechanisms of gene regulation by clastogens

**FIGURE 2:**
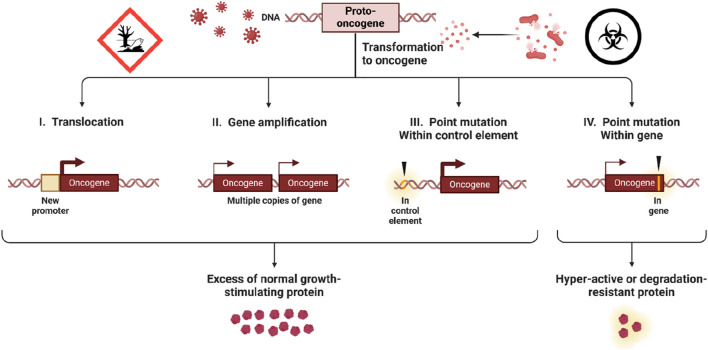
Methods of Oncogene Activation in Cancer. The diagram illustrates four mechanisms by which proto-oncogenes transform into oncogenes, contributing to cancer development. Translocation places a proto-oncogene under a new promoter, gene amplification creates multiple gene copies, and point mutations either within control elements or within the gene itself lead to excessive or hyperactive protein production. These changes result in unregulated cell growth, driving cancer progression.

The regulation of gene expression by chemical clastogens involves intricate molecular mechanisms that govern transcriptional activation, repression, and epigenetic modifications. Clastogens can influence gene expression through direct interactions with DNA, as well as through modulation of signaling pathways and transcription factors (Beyersmann and Hechtenberg, 1997). As shown in Figure 2 below, some of the key molecular mechanisms of gene regulation by clastogens include:

1. Direct DNA damage: Clastogens can directly induce DNA lesions, such as base oxidation, nitration, methylation, and single-strand or double-strand breaks. These DNA damages, if not properly repaired, can lead to mutations in the coding sequences of proto-oncogenes and tumor suppressor genes (Levin and Lilis, 2008).
2. DNA Damage Response: Clastogens induce DNA damage and activate cellular DNA damage response pathways, including the ATM/ATR-mediated DNA damage checkpoint and the p53 tumor suppressor pathway (Alhmoud et al., 2020). These signaling cascades can lead to the activation or repression of specific target genes involved in cell cycle control, DNA repair, and apoptosis (Fernandez, 2017).
3. Transcription Factor Activation: Clastogens can modulate the activity of transcription factors involved in gene regulation by altering their post-translational modifications, subcellular localization, or DNA-binding affinity (Dwarakanath et al., 2008). For example, clastogens can activate transcription factors such as NF-κB and AP-1, which regulate the expression of genes involved in inflammation, cell proliferation, and survival (Valko et al., 2006).
4. Epigenetic Modifications: Chemical clastogens can induce epigenetic modifications, such as DNA methylation and histone acetylation, that alter chromatin structure and gene expression patterns. These epigenetic changes can lead to long-lasting alterations in gene expression profiles and contribute to the development of cancer and other diseases (Kobets et al., 2019).
5. Non-coding RNA Regulation: Clastogens can influence gene expression through the regulation of non-coding RNAs, including microRNAs (miRNAs) and long non-coding RNAs (lncRNAs) (Shvedova et al., 2016). These non-coding RNAs can act as post-transcriptional regulators of gene expression by targeting mRNAs for degradation or translational repression, thereby modulating cellular responses to clastogen-induced stress (Statello et al., 2021).
6. Disruption of cell cycle checkpoints: Clastogens can impair the function of cell cycle checkpoint proteins, such as Chk1, Chk2, and the spindle assembly checkpoint proteins (Mad, Bub). This can allow cells with damaged DNA to continue dividing, leading to the propagation of genetic alterations (Reshmi, 2005).
7. Oxidative stress and signaling pathway dysregulation: Clastogens can induce oxidative stress and disrupt cellular signaling pathways, which can indirectly contribute to the activation of proto-oncogenes and the inactivation of tumor suppressor genes (Rani and Gupta, 2015).

By understanding the specific mechanisms by which chemical clastogens can activate proto-oncogenes and deactivate tumor suppressor genes, researchers and regulatory bodies can better assess the potential carcinogenic risks associated with chemical exposures and develop strategies for mitigating these risks (Singh et al., 2024b).

## 2 Impact of genomic instability on carcinogenesis

Genomic instability, characterized by an increased frequency of DNA damage, mutations, and chromosomal aberrations, is a hallmark feature of cancer. Understanding the link between clastogen-induced genomic instability and cancer development, as well as the epidemiological evidence supporting carcinogenic risk, is essential for elucidating the role of chemical exposures in cancer etiology and guiding public health interventions (Singh et al., 2021c).

### 2.1 Link between clastogen-induced genomic instability and cancer development

Chemical clastogens exert their carcinogenic effects by inducing DNA damage and genomic instability, which can promote tumorigenesis through several mechanisms. Clastogen-induced DNA damage can lead to the accumulation of mutations in oncogenes, tumor suppressor genes, and other cancer-related genes, altering their function and promoting oncogenic transformation (Basu, 2018). For example, chromosomal translocations involving proto-oncogenes such as c-Myc and Bcl-2 can result in constitutive activation of oncogenic signaling pathways, driving uncontrolled cell proliferation and survival (Shortt and Johnstone, 2012). Clastogen-induced chromosomal instability and aneuploidy can disrupt normal cell division processes, leading to the generation of genetically heterogeneous cell populations with altered karyotypes (Garribba et al., 2023). This genomic chaos can facilitate the acquisition of additional mutations and chromosomal rearrangements, fueling tumor evolution and progression (Kjeldsen, 2022). Clastogen-induced genomic instability can generate diverse genetic alterations within tumors, contributing to intra-tumoral heterogeneity and therapeutic resistance (Kumar, 2020). Tumor cells with distinct genomic profiles may exhibit differential responses to treatment modalities, leading to treatment failure and disease relapse (De Palma and Hanahan, 2012). Clastogens can impair DNA repair pathways, including base excision repair, nucleotide excision repair, and homologous recombination, compromising the cell’s ability to repair DNA damage and maintain genomic integrity (Fu et al., 2012). Persistent DNA lesions and unrepaired DNA breaks can accumulate over time, promoting the accumulation of mutations and genomic instability (Asaithamby et al., 2011). Understanding the mechanistic link between clastogen-induced genomic instability and cancer development is critical for identifying potential targets for cancer prevention and intervention strategies (Lin and Yan, 2008). By elucidating the molecular pathways through which clastogens exert their carcinogenic effects, researchers can develop novel therapeutic approaches aimed at mitigating the adverse health effects of chemical exposures (Hartwig et al., 2020).

### 2.2 Epidemiological evidence supporting carcinogenic risk

Epidemiological studies provide compelling evidence linking chemical exposures to increased cancer risk in human populations. These studies utilize various study designs, including cohort studies, case-control studies, and meta-analyses, to assess the association between chemical exposures and cancer incidence (Council et al., 2012). Some key findings from epidemiological research supporting the carcinogenic risk of clastogens include:

1. Occupational Exposures: Epidemiological studies have identified occupational exposures to chemical clastogens, such as benzene, formaldehyde, and ionizing radiation, as significant risk factors for various types of cancer, including leukemia, lymphoma, and solid tumors (Petric, 2021). Workers in industries such as petroleum refining, chemical manufacturing, and healthcare are particularly at risk of exposure to carcinogenic chemicals (Edokpolo et al., 2015).
2. Environmental Exposures: Environmental pollutants, including polycyclic aromatic hydrocarbons (PAHs), aflatoxins, and heavy metals, have been implicated in the development of cancer in exposed populations (Boffetta and Nyberg, 2003). Epidemiological studies have demonstrated associations between environmental exposures to these chemicals and increased cancer incidence, particularly in communities located near industrial facilities or hazardous waste sites (Boffetta, 2004).
3. Cancer Clusters: Epidemiological investigations of cancer clusters, defined as an unusual aggregation of cancer cases in a specific geographic area or community, have provided valuable insights into the potential carcinogenic effects of environmental exposures (Council et al., 2012). Clusters of cancer cases associated with chemical contamination of air, water, or soil have been documented in several regions worldwide, highlighting the importance of environmental monitoring and regulatory oversight (Lu et al., 2015).
4. Genetic Susceptibility: Epidemiological studies have also examined the role of genetic susceptibility factors in modifying individual susceptibility to chemical carcinogens (Caporaso, 1991). Genetic polymorphisms in genes involved in DNA repair, metabolism, and detoxification pathways can influence an individual’s ability to metabolize and eliminate carcinogenic chemicals, thereby modulating cancer risk (Gemmati et al., 2012).

### 2.3 Advances in AI/ML for chemical risk assessment

Recent advancements in artificial intelligence (AI) and machine learning (ML) have revolutionized the field of chemical risk assessment, offering powerful tools for predicting carcinogenic risk and identifying clastogen-induced genomic signatures (Wittwehr et al., 2020). Understanding the role of AI/ML in predicting carcinogenic risk and its applications in genomic signature identification is essential for harnessing the full potential of these technologies to enhance chemical risk assessment and protect public health (Kourou et al., 2015).

### 2.4 Role of artificial intelligence and machine learning in predicting carcinogenic risk

**FIGURE 3:**
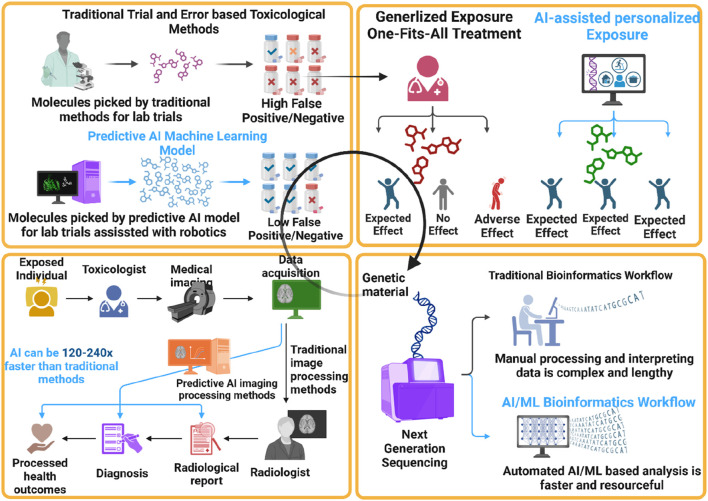
AI-ML-OMICS based Circular Toxicology Prediction Paradigm. The figure illustrates the integration of AI and machine learning (AI-ML) into the circular toxicology paradigm, showcasing significant advancements over traditional methods. It highlights how predictive AI models and robotics reduce false positives/negatives in toxicological assessments, enable personalized exposure treatments, and expedite medical imaging processing by 120–240 times. Additionally, it contrasts the manual, labor-intensive bioinformatics workflows with faster, resource-efficient AI/ML-based analyses, underscoring the transformative impact of AI-ML on improving accuracy, speed, and personalization in toxicology.

As depicted in Figure 3, AI and ML techniques play a crucial role in predicting carcinogenic risk by leveraging computational models trained on large-scale datasets comprising chemical structures, toxicity profiles, and biological responses (Lin and Chou, 2022). These techniques enable the integration of diverse data sources and the identification of complex patterns and relationships that may not be discernible through traditional statistical methods (Cavasotto and Scardino, 2022). Some key applications of AI/ML in predicting carcinogenic risk include:

1. Quantitative Structure-Activity Relationship (QSAR) Modeling: QSAR models use computational algorithms to predict the biological activity of chemical compounds based on their structural features (Vilar and Costanzi, 2012). ML techniques such as random forest, support vector machines, and neural networks are commonly employed to develop QSAR models for predicting carcinogenicity and other toxicological endpoints (Chung et al., 2023). These models can prioritize chemicals for further testing, inform regulatory decisions, and guide risk management strategies (Krewski et al., 2014).
2. Toxicogenomics and Transcriptomics: AI/ML algorithms can analyze high-throughput genomic and transcriptomic data to identify molecular signatures associated with chemical exposure and toxicity (Kleinstreuer et al., 2021). By integrating gene expression profiles with toxicological endpoints, such as carcinogenicity and genotoxicity, researchers can elucidate the mechanisms of chemical-induced toxicity and identify biomarkers indicative of adverse health effects (Fan, 2014).
3. High-Throughput Screening (HTS) Assays: HTS assays generate large datasets comprising chemical screening results and biological responses across diverse cellular and molecular endpoints. AI/ML approaches can analyze HTS data to prioritize chemicals for further testing, identify structure-activity relationships, and predict potential hazards and risks. These predictive models can accelerate the chemical risk assessment process and reduce the need for costly and time-consuming animal testing (Borrel et al., 2020).
4. Data Integration and Decision Support Systems: AI/ML technologies facilitate the integration of heterogeneous data sources, including chemical databases, toxicological assays, and omics datasets, to generate comprehensive risk assessment frameworks (Martínez-García and Hernández-Lemus, 2022). Decision support systems powered by AI/ML algorithms can assist regulatory agencies, industry stakeholders, and public health officials in evaluating chemical hazards, establishing exposure limits, and implementing risk management measures (Ganesh and Kalpana, 2022).

By connecting the predictive capabilities of AI and ML, researchers can enhance the efficiency, accuracy, and scalability of chemical risk assessment, ultimately improving public health outcomes and reducing the burden of chemical-related diseases (Oki et al., 2017).

### 2.5 Applications of AI/ML in identifying clastogen-induced genomic signatures

AI and ML techniques are increasingly being employed to identify clastogen-induced genomic signatures, molecular markers, and biomarkers associated with DNA damage and genomic instability. These approaches enable the discovery of novel biomarkers indicative of clastogen exposure, elucidation of underlying mechanisms of genotoxicity, and development of predictive models for assessing carcinogenic risk. Some key applications of AI/ML in identifying clastogen-induced genomic signatures include:

1. Genomic Profiling and Molecular Signatures: AI/ML algorithms can analyze high-dimensional genomic datasets, such as DNA sequencing, gene expression, and epigenetic profiles, to identify characteristic patterns and signatures associated with clastogen exposure (Vilhekar and Rawekar, 2024). These molecular signatures may include gene expression changes, DNA methylation patterns, and chromosomal aberrations indicative of genotoxicity and carcinogenic risk (Rauschert et al., 2020).
2. Integration of Multi-Omics Data: AI/ML approaches facilitate the integration of multi-omics data, including genomics, transcriptomics, proteomics, and metabolomics, to provide a comprehensive view of clastogen-induced genomic alterations and molecular responses (Cai et al., 2022). Integrative analyses enable the identification of molecular networks, pathways, and biological processes perturbed by clastogen exposure, aiding in the elucidation of underlying mechanisms of genotoxicity (Eisenbrand et al., 2002).
3. Predictive Modeling and Risk Assessment: AI/ML algorithms can develop predictive models for assessing the carcinogenic risk of chemical clastogens based on genomic signatures and molecular endpoints (Limbu and Dakshanamurthy, 2022). These models can prioritize chemicals for further testing, predict carcinogenic outcomes, and inform regulatory decisions regarding chemical safety and exposure limits (Hartung, 2023a). By leveraging computational modeling and machine learning techniques, researchers can accelerate the identification of clastogen-induced genomic signatures and improve risk assessment accuracy (Staff, 2021).
4. Biomarker Discovery and Validation: AI/ML approaches enable the discovery and validation of biomarkers indicative of clastogen exposure and genotoxicity. By analyzing large-scale omics datasets from exposed populations and experimental models, researchers can identify candidate biomarkers associated with DNA damage, chromosomal instability, and carcinogenic risk. These biomarkers can serve as early indicators of clastogen-induced genotoxicity and facilitate early intervention and preventive measures (Demir Karaman and Işık, 2023).

AI and ML technologies offer powerful tools for identifying clastogen-induced genomic signatures and predicting carcinogenic risk (Singh et al., 2023b). By leveraging computational modeling, high-throughput data analysis, and integrative omics approaches, researchers can enhance our understanding of clastogen-induced genotoxicity, improve chemical risk assessment methodologies, and ultimately safeguard public health (Wei et al., 2023). These AI/ML-driven approaches can help researchers and regulatory bodies to develop predictive models for identifying chemicals with a high likelihood of inducing genomic instability and carcinogenic potential (Kourou et al., 2021; Cavasotto and Scardino, 2022). Computational approaches further elucidate the molecular mechanisms by which chemical clastogens contribute to the development of cancer, enabling the design of targeted interventions and preventive strategies (Kourou et al., 2015). For oncologist, AI can establish biomarkers and signatures that can be used for early detection, risk assessment, and monitoring of the health impacts associated with chemical exposures (Cavasotto and Scardino, 2022; Yang and Kar, 2023). Computational field may accelerate the screening and prioritization of chemicals for further toxicological evaluation, optimizing the use of resources and reducing the reliance on animal testing (Vatansever et al., 2021; Cavasotto and Scardino, 2022). Table 1 summarizes various tools available for toxicity prediction, detailing their type, unique features, prediction principles, reliability pointers, and URLs for further information. The tools range from free to commercial and utilize diverse methodologies, including machine learning models, QSAR, read-across, and expert rule-based systems. They are designed for different purposes, such as screening-level assessments and regulatory submissions, with validated performance metrics ensuring reliability.

**TABLE 1:** An Overview of Toxicity Prediction Tools for Carcinogenicity, Genotoxicity and Mutagenicity assessment.

Tool name	Type	Unique features	Prediction principles	Reliability pointers	URL
CarcinoPred-EL	Free	Ensemble of 3 ML models (SVM, RF, XGB)	Uses 7 types of molecular fingerprints and 3 types of physicochemical descriptors	Validated on external test set, AUC-ROC >0.75	https://ccsb.scripps.edu/carcinopred-el/
Ames Mutagenicity Predictor	Free	Naïve Bayes classification models	Utilizes molecular descriptors and fingerprints	Validated on external test set, accuracy >80%	https://github.com/zhanghuiyi/Ames-Mutagenicity-Predictor
QSAR Toolbox	Free	Integrated platform for toxicity prediction	Combines QSAR, read-across, and expert rule-based systems	Follows OECD principles for QSAR model development	https://www.oecd.org/chemicalsafety/risk-assessment/oecd-qsar-toolbox.htm
Derek Nexus	Commercial	Knowledge-based expert system for toxicity prediction	Uses structural alerts and expert rules	Transparent reasoning, suitable for regulatory submissions	https://www.lhasalimited.org/products/derek-nexus.htm
Leadscope Model Applier	Commercial	Comprehensive suite of QSAR models	Utilizes molecular descriptors, fingerprints, and structural alerts	Validated on diverse datasets, transparent model explanations	https://www.leadscope.com/model_applier/
ADMET Predictor	Commercial	Integrates QSAR, expert rules, and ML models	Predicts various ADMET endpoints including genotoxicity	Robust model development following regulatory guidelines	https://www.simulations-plus.com/software/admetpredictor/
ToxGPS	Commercial	Combines QSAR, read-across, and expert knowledge	Predicts various toxicity endpoints including carcinogenicity	Transparent model interpretability, suitable for regulatory use	https://www.lhasalimited.org/products/toxgps.htm
EPA Toxicity Estimation Software Tool (TEST)	Free	QSAR-based tool for toxicity prediction	Utilizes multiple QSAR methodologies (e.g., hierarchical clustering, FDA)	Freely available, suitable for screening-level assessments	https://www.epa.gov/chemical-research/toxicity-estimation-software-tool-test
VEGA	Free	Integrated platform for toxicity prediction	Combines QSAR, read-across, and expert rule-based systems	Follows OECD principles, provides model performance metrics	https://www.vegahub.eu/
OCHEM	Free	Web-based platform for QSAR modeling	Allows users to build, validate, and apply QSAR models	Supports various ML algorithms, provides model performance metrics	https://ochem.eu/
ChemTunes + TOX	Commercial	Integrates QSAR, read-across, and expert knowledge	Predicts various toxicity endpoints including genotoxicity	Robust model development, transparent model explanations	https://www.compudrug.com/chemtunes-tox
ToxEval	Free	Screening-level tool for toxicity assessment	Utilizes structure-activity relationship rules and read-across	Freely available, suitable for initial hazard identification	https://www.epa.gov/chemical-research/toxevaluator-software-application
TIMES-SS	QSAR	Free	Statistical model, ensemble learning	Predicts genotoxicity and carcinogenicity based on	https://pubs.acs.org/doi/10.1021/ci050150r
Chembench	QSAR	Free, web-based	Integrates various QSAR models	Offers model validation and comparison tools	http://chembench.mml.unc.edu/

Based on recent studies and evaluations, the accuracy of the tools listed in Table One varies based on their underlying methodologies and the quality of data used for training. For instance, while tools like CarcinoPred-EL and Ames Mutagenicity Predictor have demonstrated robust predictive performance in external validation studies (with AUC values exceeding 0.75 and accuracy rates above 80%, respectively), limitations exist in their application. These limitations include a reliance on specific chemical classes or structural features, which may not generalize well across diverse datasets (Honma et al., 2019; Zhang et al., 2017).

Rule-based or expert-based systems, such as Derek Nexus, utilize established toxicological knowledge and structural alerts to predict toxicity, offering transparency in their reasoning. This approach can be particularly beneficial in regulatory contexts where understanding the rationale behind predictions is crucial. In contrast, purely data-driven models, like those based on machine learning techniques, can capture complex patterns but often lack interpretability, which poses challenges in regulatory acceptance (Cronin et al., 2022).

Furthermore, the integration of both methodologies—expert knowledge with data-driven approaches—can yield improved predictive accuracy and reduce the likelihood of errors. Recent studies have suggested that hybrid models that combine the strengths of both systems can enhance performance in toxicity predictions while maintaining interpretability (Link et al., 2022).

### 2.6 Computational tools based on OMICS

Computational tools based on OMICS are crucial for uncovering the molecular mechanisms of carcinogenicity by integrating large-scale biological data, enabling more accurate predictions of chemical toxicity and enhancing risk assessment, and several computational approaches and tools currently utilize OMICS datasets (e.g., genomics, transcriptomics, proteomics, metabolomics) for predicting carcinogenicity. These tools leverage the power of AI/ML models to handle the complexity and scale of OMICS data, providing more accurate and biologically informed predictions (Toussaint et al., 2024). Below are some key advancements to the OMICS section widening the horizon of chemical carcinogenic assessment:

1. Integration of Multi-Omics Data: Tools that integrate multiple OMICS layers (genomics, transcriptomics, proteomics) to provide a systems biology view of carcinogenicity, offering deeper insights into the molecular mechanisms driving genomic instability and cancer risk. Examples include platforms like MetaboAnalyst and multi-omics AI platforms that enable the integration of diverse datasets for toxicity prediction (Badwan et al., 2023).
2. AI/ML Models for OMICS-Based Toxicity Prediction: AI-driven models such as Deep Neural Networks (DNNs) and Random Forest (RF) classifiers that utilize OMICS datasets (e.g., gene expression, methylation data) to predict chemical-induced carcinogenicity. These models have demonstrated high predictive accuracy for classifying chemicals based on carcinogenic potential by integrating molecular-level information from OMICS data (Sahu et al., 2022).
3. Toxicogenomics Data Interpretation Tools: Tools like TG-GATEs (Toxicogenomics Project-Genomics Assisted Toxicity Evaluation System) and ToxCast that combine transcriptomics data with machine learning models for evaluating gene expression changes upon chemical exposure, facilitating the identification of biomarkers and pathways associated with cancer risk (Jia et al., 2023).
4. Pathway-Based Analysis Tools: Platforms such as Pathway Studio and IPA (Ingenuity Pathway Analysis), which enable the integration of OMICS data with AI/ML for pathway and network analysis. These tools predict carcinogenic risk by identifying disrupted pathways and molecular signatures linked to chemical exposure (Nagasaki et al., 2009).
5. Proteomics-Based Predictive Models: Advances in proteomics have enabled the use of computational tools that predict chemical toxicity based on protein expression profiles. Tools like ProTox-II utilize AI to analyze protein-level changes to assess chemical carcinogenicity, focusing on post-translational modifications and protein-protein interaction networks (Banerjee et al., 2018).
6. Epigenetic Modulation and Carcinogenicity: Current models also include AI/ML models that incorporate epigenomic data (such as DNA methylation patterns and histone modifications) to predict carcinogenic outcomes. These models offer an additional layer of predictive power by accounting for epigenetic changes that influence gene expression and genomic instability (Arslan et al., 2021).
7. Challenges and opportunities: Navigating the landscape of chemical risk assessment involves addressing various challenges while leveraging opportunities to enhance methodologies and technologies. Understanding the limitations of current approaches and envisioning future directions for integrating AI/ML and omics technologies can guide efforts toward more effective and comprehensive risk assessment strategies (Hartung, 2023a).

### 2.7 Limitations of current approaches in chemical risk assessment: an overview of dose-exposure data significance

**FIGURE 4:**
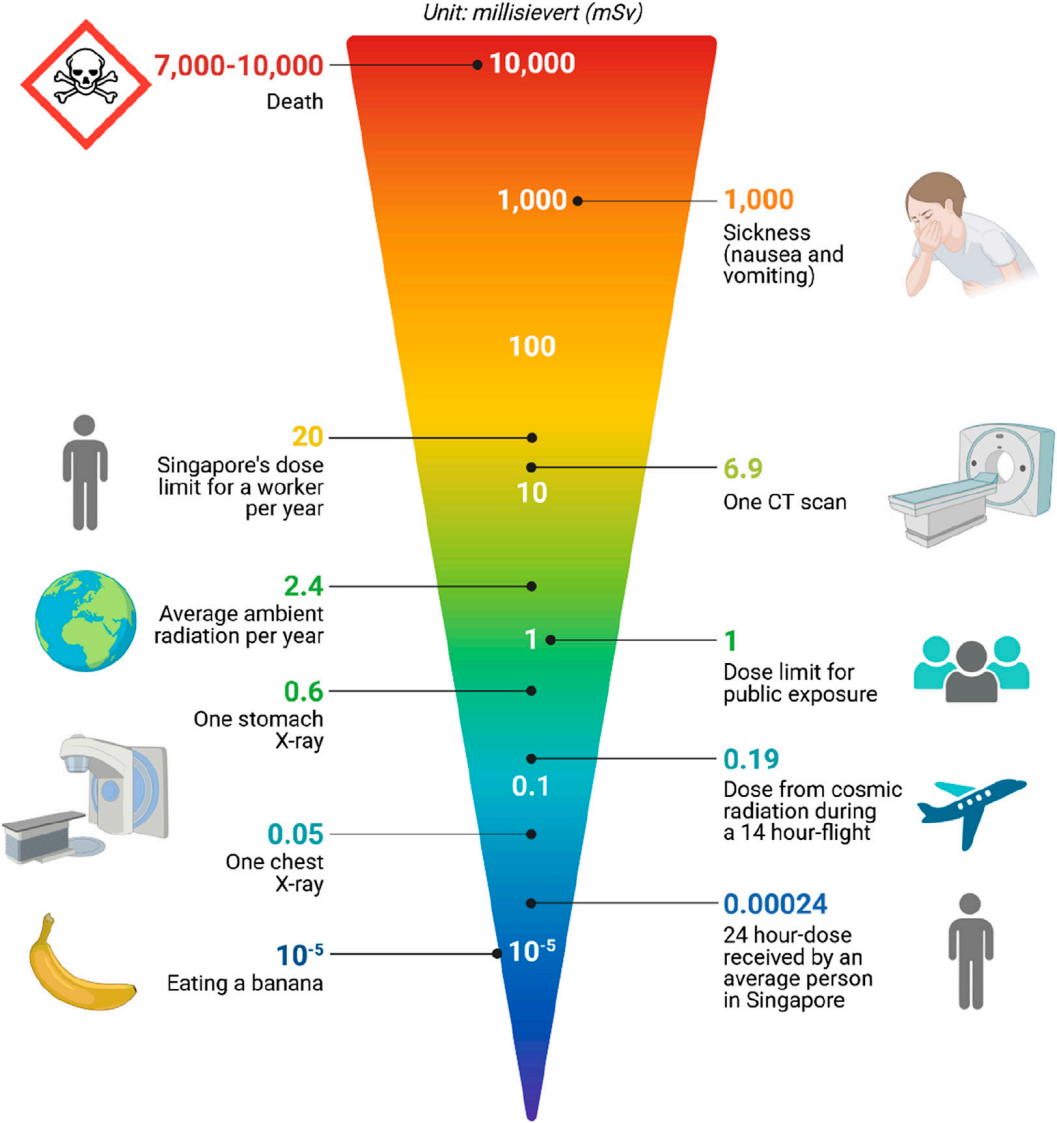
Distribution of Radiology Doses Across Different Imaging Modalities This figure shows the comparative radiation doses received by patients from different radiological procedures, such as X-rays, CT scans, MRI scans, and PET scans. The bar chart details the average dose in millisieverts (mSv) for each modality, emphasizing the variability in radiation exposure and its implications for patient safety. It also highlights guidelines and thresholds for safe exposure levels to inform clinical decision-making and minimize radiation risks.

Figure 4 below illustrates the critical role of data in understanding and managing DSE (Digital Systems Exposure) scenarios (Singh et al., 2020b). It highlights various data sources, including environmental monitoring, personal exposure records, and health outcomes, and their integration into a comprehensive risk assessment framework. The diagram underscores the importance of accurate and timely data collection to enhance predictive modeling, policy-making, and public health interventions in exposure scenarios.

1. Dose Exposure Data Availability and Quality: One of the primary limitations in chemical risk assessment is the availability and quality of data, particularly for assessing long-term health effects and low-dose exposures. Limited datasets and inconsistencies in data quality hinder the development of robust predictive models and risk assessment frameworks (Whaley et al., 2016).
2. Complexity of Toxicity Mechanisms: Chemical toxicity involves complex interactions between xenobiotics and biological systems, encompassing diverse molecular pathways and cellular responses. Current approaches often struggle to capture the multifaceted nature of toxicity mechanisms, leading to oversimplified models and inaccurate predictions (Rieger et al., 2002).
3. Species Extrapolation and Uncertainty: Extrapolating toxicity data from animal models to humans poses challenges due to species-specific differences in metabolism, physiology, and sensitivity to chemical exposures. Uncertainties in extrapolation methods and reliance on default assumptions contribute to uncertainty in risk assessment outcomes (Van Beugen, 2019).
4. Regulatory Compliance and Resource Constraints: Regulatory requirements for chemical risk assessment often necessitate extensive data generation, testing, and evaluation, imposing significant time and resource burdens on regulatory agencies, industry stakeholders, and researchers. Compliance with regulatory standards may be challenging, particularly for emerging chemicals and novel compounds lacking sufficient toxicological data (Lewis et al., 2007).

## 4 Future directions and opportunities for integration of AI/ML and omics

1. Data Integration and Multi-Omics Approaches: Future directions in chemical risk assessment involve integrating diverse omics datasets, including genomics, transcriptomics, proteomics, and metabolomics, to provide a comprehensive view of molecular responses to chemical exposures. Multi-omics approaches enable the identification of molecular signatures, pathways, and networks associated with toxicity mechanisms, enhancing the predictive power of risk assessment models (Buesen et al., 2017).
2. Advanced Machine Learning and AI Techniques: Leveraging advanced machine learning algorithms, such as deep learning, reinforcement learning, and Bayesian networks, holds promise for enhancing predictive modeling and risk assessment capabilities (Adeoye, 2023). These techniques enable the extraction of complex patterns and relationships from large-scale omics datasets, facilitating the development of more accurate and interpretable predictive models for chemical toxicity (Cavasotto and Scardino, 2022).
3. Toxicity Pathway Analysis and Systems Biology: Future directions in chemical risk assessment involve adopting systems biology approaches to elucidate toxicity pathways and molecular mechanisms underlying adverse health effects (Cote et al., 2016). Integrating omics data with pathway analysis tools enables the reconstruction of molecular networks and pathways affected by chemical exposures, providing insights into mode of action, dosedosdosedose-response relationships, and biological relevance (Aguayo-Orozco et al., 2019).
4. Predictive Toxicology and Computational Models: Advancements in predictive toxicology and computational modeling offer opportunities for developing mechanistically informed models of chemical toxicity (Kavlock et al., 2008). Integrating omics data with computational models allows for the refinement of toxicity predictions, identification of key biomarkers, and prioritization of chemicals for further testing, validation, and regulatory decision-making (Wang et al., 2021).

By addressing the limitations of current approaches and embracing opportunities for integration of AI/ML and omics technologies, researchers and stakeholders can advance the field of chemical risk assessment, improve public health protection, and promote sustainable development.

### 4.1 Case studies and applications highlighting successful applications of ai/ml and omics in chemical risk assessment

Illustrating successful applications of AI/ML and omics in chemical risk assessment provides valuable insights into the practical utility and efficacy of integrative approaches. Case studies demonstrating the synergy between AI/ML and omics technologies offer tangible examples of how these methodologies can enhance predictive modeling, toxicity assessment, and risk management strategies.

In toxicity prediction for environmental chemicals, AI/ML-based predictive models, integrated with omics data, have been successfully applied to assess the toxicity of environmental chemicals (Jeong and Choi, 2022). For example, researchers have developed QSAR models combining chemical descriptors with transcriptomics data to predict the toxicity of industrial chemicals, pesticides, and pharmaceuticals, enabling rapid screening and prioritization of chemicals for regulatory evaluation (Ghosh et al., 2020). In drug safety assessment in pharmaceutical industry, Pharmaceutical companies leverage AI/ML and omics technologies for drug safety assessment and toxicity profiling during drug development (Niazi, 2023). Integrated omics approaches, such as toxicogenomics and metabolomics, enable the identification of drug-induced adverse effects, elucidation of toxicity mechanisms, and early detection of potential safety concerns, leading to informed decision-making and optimization of drug candidates (Beilmann et al., 2019). The environmental monitoring and biomonitoring programs utilize AI/ML algorithms and omics-based biomarkers to assess chemical exposures and environmental health risks (Scott et al., 2023). For instance, transcriptomics and proteomics profiling of aquatic organisms exposed to pollutants enable the identification of molecular biomarkers indicative of environmental stress and contaminant effects, informing regulatory decisions and ecosystem management strategies (Hampel et al., 2016). In precision toxicology and personalized risk assessment, AI/ML-driven precision toxicology approaches integrate omics data with individual-level exposure information to tailor risk assessment and management strategies to specific populations or subgroups (Singh et al., 2023a). By accounting for inter-individual variability in genetic susceptibility, metabolic phenotype, and environmental factors, personalized risk assessment frameworks enhance the accuracy and relevance of toxicity predictions, supporting targeted intervention and prevention efforts (Pedrete et al., 2016).

### 4.2 Case studies demonstrating the utility of integrative approaches

1. Integrative Toxicity Assessment of Nanomaterials: Researchers employed a multi-omics approach combining genomics, transcriptomics, and proteomics to evaluate the toxicity of engineered nanomaterials (ENMs) (Smythers, 2023). By integrating omics data with computational modeling and toxicological assays, the study elucidated the mechanisms of ENM-induced cytotoxicity, oxidative stress, and inflammation, providing insights into nanomaterial safety and regulatory implications (Panagiotou and Taboureau, 2012).
2. Development of Predictive Models for Chemical Carcinogenicity: A collaborative effort between academia, industry, and regulatory agencies utilized AI/ML-based QSAR models integrated with genomics and transcriptomics data to predict the carcinogenic potential of industrial chemicals and environmental contaminants (Anklam et al., 2022). The predictive models, validated using *in vitro* and *in vivo* assays, demonstrated high accuracy in identifying chemical carcinogens and non-carcinogens, enabling informed risk assessment and regulatory decision-making (Tcheremenskaia et al., 2019).
3. Environmental Exposure Assessment Using Biomarkers: Biomonitoring studies in occupational and environmental settings employed omics-based biomarkers, such as gene expression signatures, protein profiles, and metabolite patterns, to assess chemical exposures and health risks (Ge et al., 2013). By correlating omics data with exposure levels and health outcomes, these studies identified molecular indicators of exposure, effect, and susceptibility, supporting evidence-based risk assessment and intervention strategies (DeBord et al., 2015).
4. Translational Applications in Public Health: Integrative approaches combining AI/ML and omics technologies have translational applications in public health surveillance, disease prevention, and regulatory policy (Barghash et al., 2021; Rai et al., 2023b). For example, population-based studies incorporating omics-based biomarkers and exposure data enable the identification of environmental health disparities, assessment of chemical-related disease burden, and development of targeted interventions to protect vulnerable populations (Everson and Marsit, 2018).

By showcasing successful case studies and applications of AI/ML and omics in chemical risk assessment, researchers and stakeholders can demonstrate the real-world impact and transformative potential of integrative approaches for advancing public health protection and environmental stewardship (Amiri et al., 2023).

## 5 Ethical and regulatory implications

Addressing the ethical considerations and regulatory perspectives surrounding the use of AI/ML in risk assessment, as well as the integration of omics data into risk assessment frameworks, is crucial for ensuring the responsible and transparent application of these technologies in safeguarding public health and environmental safety.

### 5.1 Ethical considerations surrounding the use of AI/ML in risk assessment

By addressing ethical considerations surrounding the use of AI/ML in risk assessment and regulatory perspectives on incorporating omics data into risk assessment frameworks, policymakers, researchers, and stakeholders can promote responsible innovation, transparency, and accountability in chemical risk assessment practices, ultimately advancing public health and environmental safety (Bruschi and Diomede, 2023). The storage and use of whole-genome sequencing (WGS) tumor data present significant ethical challenges, particularly in terms of privacy, consent, and data security. One key example is the ongoing debate over the ethical management of genomic data within large-scale cancer research initiatives like The Cancer Genome Atlas (TCGA). While TCGA has enabled groundbreaking discoveries in cancer genomics, it has also raised concerns about the potential for re-identification of patients, despite anonymization efforts (Horton and Lucassen, 2023).

Regulatory frameworks such as the European General Data Protection Regulation (GDPR) have sought to address these issues by enforcing strict guidelines on the collection, storage, and use of personal genomic data. GDPR mandates explicit informed consent for the use of such data in research and imposes penalties for breaches, making it a critical reference point for ensuring ethical compliance in WGS data handling (Shabani and Borry, 2018). Additionally, the Health Insurance Portability and Accountability Act (HIPAA) in the U.S. governs the secure storage of health data, including WGS data, providing further safeguards against unauthorized access and ensuring patient privacy (Thompson, 2020).

1. Data Privacy and Confidentiality: Ethical concerns arise regarding the collection, storage, and sharing of sensitive biological and health data in AI/ML-based risk assessment (Federico and Trotsyuk, 2024). Ensuring data privacy, confidentiality, and informed consent is essential to protect individuals’ rights and mitigate the risk of unauthorized access, misuse, or discrimination based on personal information (Hlávka, 2020).
2. Bias and Fairness: AI/ML algorithms may inadvertently perpetuate biases and inequities present in training data, leading to biased predictions and discriminatory outcomes. Ethical considerations include addressing algorithmic bias, ensuring fairness and transparency in decision-making processes, and promoting equity and inclusivity in risk assessment practices (Leslie et al., 2024; Shuford, 2024).
3. Accountability and Transparency: Transparency and accountability are essential principles in AI/ML-based risk assessment to enable scrutiny, validation, and reproducibility of predictive models and decisions. Ethical guidelines advocate for transparent reporting of methodologies, data sources, model assumptions, and limitations to foster trust, accountability, and responsible use of AI/ML technologies (Drabiak et al., 2023).
4. Human Oversight and Interpretability: Maintaining human oversight and interpretability in AI/ML-driven risk assessment is critical to ensure that algorithmic predictions align with domain expertise, ethical principles, and societal values. Ethical frameworks emphasize the importance of human judgment, critical reasoning, and ethical reasoning in guiding decision-making processes and mitigating the potential for unintended consequences or harm (Lo Piano, 2020).

### 5.2 Regulatory perspectives on incorporating omics data into risk assessment frameworks

• Regulatory bodies have begun incorporating AI models into their chemical risk assessment workflows. For instance, the U.S. Environmental Protection Agency (EPA) has implemented the Toxicity Estimation Software Tool (TEST), which uses machine learning algorithms to predict toxicity endpoints, including carcinogenicity, based on chemical structure data (Hartung, 2023b). Similarly, the OECD’s QSAR Toolbox facilitates regulatory compliance by allowing member countries to use AI-driven models for hazard identification and risk assessments. These tools are designed to reduce the need for animal testing by providing robust *in silico* predictions for regulatory submissions (Dimitrov et al., 2016).

Moreover, the European Food Safety Authority (EFSA) has utilized AI-based models in its food safety assessments, particularly for prioritizing chemicals in terms of potential health risks. EFSA’s work in developing AI-enhanced approaches for pesticide risk assessments represents a significant step toward automating and refining regulatory toxicology processes (Blümmel et al., 2023). The primary challenge faced by these agencies in adopting AI is ensuring the transparency, reproducibility, and validation of AI models, which remain key factors in regulatory decision-making (Cavalli et al., 2019). Below are some key points addressing adaption to *in silico* approaches to regulatory perspectives-

1. Data Quality and Standardization: Regulatory agencies emphasize the importance of data quality, reliability, and standardization in incorporating omics data into risk assessment frameworks. Guidelines for omics-based biomarker validation, assay reproducibility, and data quality control aim to ensure the integrity and validity of experimental results and facilitate regulatory acceptance and decision-making (Marx-Stoelting et al., 2015).
2. Validation and Qualification: Regulatory perspectives on omics data integration prioritize the validation and qualification of biomarkers and predictive models for risk assessment purposes. Validation studies assess the sensitivity, specificity, accuracy, and robustness of omics-based assays and algorithms in predicting toxicity outcomes, informing regulatory decisions on biomarker qualification and acceptance criteria (Omenn et al., 2012).
3. Risk Characterization and Uncertainty Assessment: Regulatory frameworks for integrating omics data into risk assessment emphasize the importance of risk characterization, uncertainty assessment, and evidence-based decision-making. Omics-based biomarkers and predictive models should be evaluated in the context of exposure assessment, hazard identification, dose-response analysis, and risk characterization to inform risk management strategies and regulatory actions (Van Aggelen et al., 2010).
4. Translational Research and Application: Regulatory agencies support translational research and application of omics technologies in risk assessment to enhance public health protection and regulatory decision-making. Collaborative efforts between academia, industry, and regulatory stakeholders facilitate the development, validation, and implementation of omics-based assays, biomarkers, and predictive models for regulatory use, promoting innovation and scientific advancement in chemical safety assessment (Matthews et al., 2016). One prominent example of collaboration between academia, industry, and regulatory stakeholders is the EU-ToxRisk project, a European Commission-funded initiative aimed at advancing non-animal testing strategies for chemical safety assessment. The project integrates AI and OMICS technologies to develop predictive models that assess the toxicological effects of chemicals on human health. Regulatory bodies such as the European Chemicals Agency (ECHA) and EFSA are actively involved in validating these models for regulatory use. Additionally, the U.S. FDA has partnered with several academic institutions to apply AI in toxicogenomics, particularly for drug safety assessments. This collaboration has led to the development of AI tools that analyze gene expression data from toxicogenomics studies to predict adverse drug reactions, aiding regulatory agencies in early-stage drug approval processes. These efforts have successfully demonstrated how AI can streamline the assessment of chemical safety while reducing reliance on animal testing (Smirnova et al., 2018; Sillé et al., 2020).

### 5.3 COSMIC signature database and causal inference in carcinogenesis

The COSMIC (Catalogue Of Somatic Mutations In Cancer) signature database is an invaluable resource for understanding the mutational processes underlying cancer. It catalogs mutational signatures—patterns of mutations associated with specific carcinogenic processes—which can provide insights into the etiology of different cancer types. These signatures, derived from large-scale sequencing data, help infer causal links between environmental exposures and cancer (Alexandrov et al., 2020).

One notable example is the identification of signature 7, which has been strongly linked to UV radiation exposure. This signature, characterized by C > T transitions in dipyrimidine contexts, has been instrumental in confirming UV radiation as a major driver of skin cancer, particularly melanoma. A recent study utilizing the COSMIC database demonstrated population-specific differences in UV-induced mutational burden, further advancing our understanding of how environmental factors like UV exposure contribute to cancer risk in different populations​ (D’Orazio et al., 2013).

Similarly, COSMIC signatures have been used to explore the origins of liver cancer, revealing associations between specific mutational patterns and aflatoxin exposure, a known risk factor for hepatocellular carcinoma. The ability to pinpoint these mutational signatures provides a powerful tool for causal inference, enabling researchers to identify carcinogenic agents and their impact on specific tissues. This approach not only advances our knowledge of cancer etiology but also aids in the development of targeted prevention strategies and therapeutic interventions (Phillips, 2018).

By incorporating insights from COSMIC mutational signatures, one can enhance the precision of carcinogenicity assessments and further our understanding of the environmental and genetic factors that contribute to cancer development (Bindal et al., 2011).

## 6 Summary of key findings, outlook and conclusion

This review has explored the intersection of AI/ML and omics technologies in chemical risk assessment, highlighting their synergistic potential to revolutionize predictive modeling, toxicity assessment, and risk management strategies. From elucidating toxicity mechanisms to predicting adverse health outcomes, integrating AI/ML and omics offers a comprehensive approach to understanding chemical exposures and safeguarding public health (Krause et al., 2021).

Throughout this review, several key findings have emerged:

1. Integration of AI/ML and Omics: Combining AI/ML algorithms with omics technologies enables the analysis of large-scale biological data, including genomics, transcriptomics, proteomics, and metabolomics, to elucidate molecular responses to chemical exposures. Integrative approaches facilitate the identification of molecular signatures, pathways, and networks associated with toxicity mechanisms, enhancing the predictive power and accuracy of risk assessment models.
2. Advancements in Predictive Modeling: AI/ML-driven predictive modeling techniques, such as QSAR models, deep learning algorithms, and ensemble methods, offer powerful tools for predicting genotoxicity, mutagenicity, and carcinogenicity of chemical compounds (Mone et al., 2023). By leveraging omics data and computational modeling, researchers can develop mechanistically informed models that prioritize chemicals for testing, inform regulatory decisions, and guide cancer prevention strategies (Singh et al., 2018; Singh et al., 2024c).
3. Translational Applications and Case Studies: Successful applications of AI/ML and omics in chemical risk assessment have been demonstrated across various sectors, including pharmaceuticals, environmental monitoring, and public health. Case studies showcasing the utility of integrative approaches provide tangible examples of how these technologies can enhance predictive modeling, toxicity assessment, and risk management practices in real-world settings.

### 6.1 Future perspectives for advancing chemical risk assessment and cancer prevention

**FIGURE 5:**
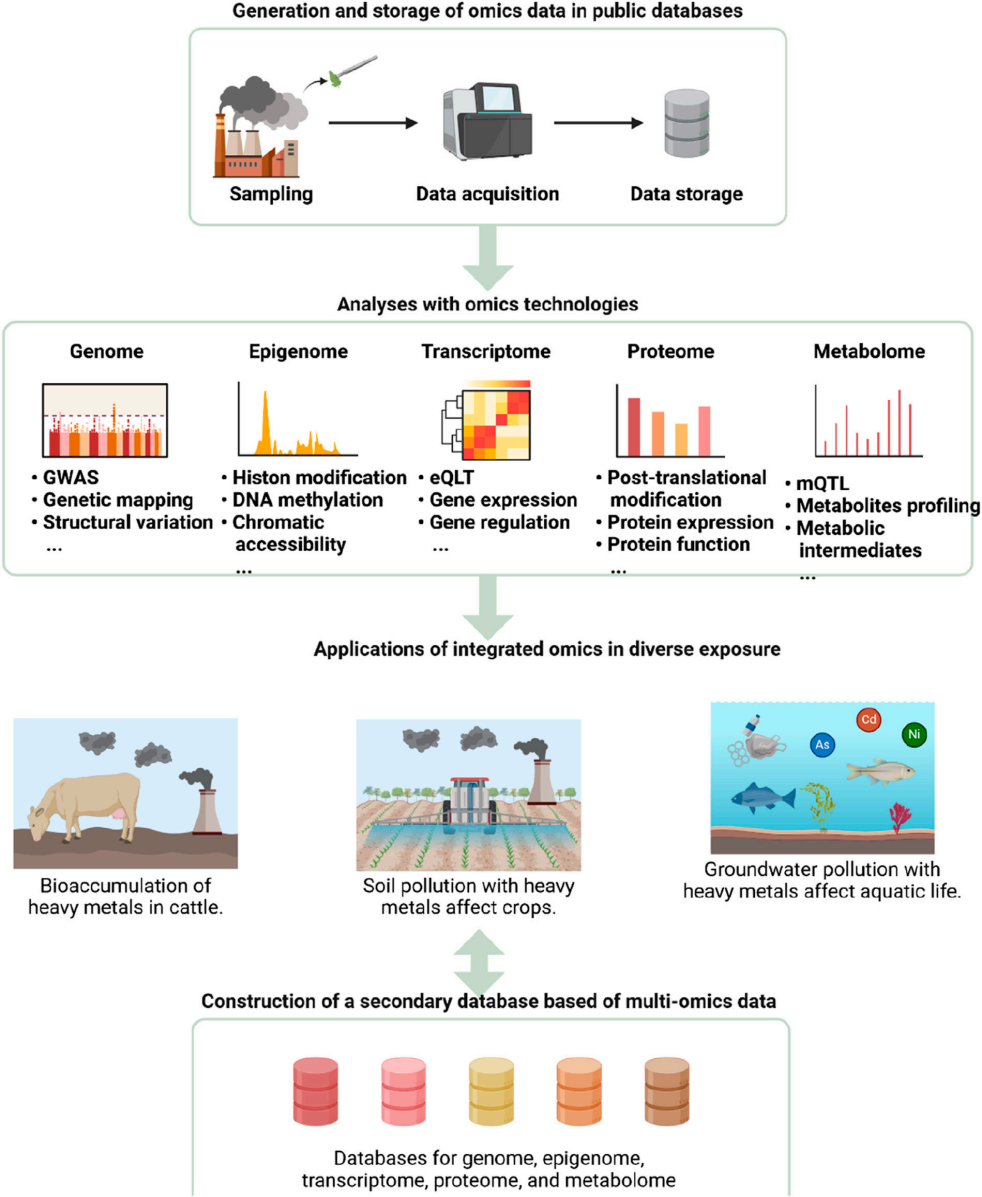
Application of Integrated Omics Technologies in Environmental Exposure Studies. The diagram outlines the workflow and applications of integrated omics technologies in analyzing environmental exposures. The process begins with sampling, data acquisition, and storage in public databases. Various omics technologies, including genomics, epigenomics, transcriptomics, proteomics, and metabolomics, are then applied to analyze the data. These analyses encompass genome-wide association studies (GWAS), epigenetic modifications, gene expression regulation, protein expression and function, and metabolite profiling. The integrated omics data are utilized to study the bioaccumulation of heavy metals in cattle, soil pollution affecting crops, and groundwater contamination impacting aquatic life. Ultimately, a secondary database based on multi-omics data is constructed, facilitating comprehensive studies on genome, epigenome, transcriptome, proteome, and metabolome interactions.

Looking ahead, several future perspectives emerge for advancing chemical risk assessment and cancer prevention:

1. Data Integration and Multi-Omics Approaches: Further integration of diverse omics datasets and development of multi-omics approaches will enhance our understanding of chemical toxicity mechanisms and enable more accurate prediction of adverse health outcomesContinued efforts to standardize data formats, improve data sharing practices, and develop interoperable platforms will facilitate data integration and cross-disciplinary collaboration (Del Giudice et al., 2023; Shahrajabian and Sun, 2023).
2. Advanced Machine Learning Techniques: Continued advancements in machine learning algorithms, including deep learning, reinforcement learning, and Bayesian networks, hold promise for improving predictive modeling and toxicity assessment as shown in Figure 5. Future research should focus on developing interpretable and explainable AI models that provide insights into the biological relevance and mechanistic underpinnings of predictive outcomes (Niazi and Mariam, 2023).
3. Translational Research and Policy Implementation: Bridging the gap between research and policy implementation is essential for translating scientific advancements into actionable interventions and regulatory decisions. Collaborative efforts between academia, industry, regulatory agencies, and public health stakeholders will facilitate the adoption of AI/ML and omics technologies in risk assessment practices, promoting evidence-based policy development and cancer prevention strategies (Olswang and Prelock, 2015).

The integration of AI/ML and omics technologies offers transformative opportunities for advancing chemical risk assessment and cancer prevention efforts. By harnessing the predictive capabilities of these technologies, researchers and policymakers can enhance public health protection, mitigate environmental risks, and promote sustainable development for future generations.
